# The UK vaccine innovation pathway

**DOI:** 10.1093/immadv/ltaf032

**Published:** 2025-11-15

**Authors:** Sarah Danson, Robert P Jones, Kristina Duggleby, Gillian Rosenberg, Matthew Hallsworth, Maria Koufali

**Affiliations:** Division of Clinical Medicine, School of Medicine and Population Health, University of Sheffield, Sheffield, United Kingdom; Department of Oncology, Sheffield Teaching Hospitals NHS Foundation Trust, Sheffield, United Kingdom; UK Vaccine Innovation Pathway, National Institute for Health and Care Research, London, United Kingdom; UK Vaccine Innovation Pathway, National Institute for Health and Care Research, London, United Kingdom; Department of Molecular and Clinical Cancer Medicine, Institute of Systems, Molecular and Integrative Biology, University of Liverpool, Liverpool, United Kingdom; UK Vaccine Innovation Pathway, National Institute for Health and Care Research, London, United Kingdom; NHS Cancer Programme, NHS England, London, United Kingdom; UK Vaccine Innovation Pathway, National Institute for Health and Care Research, London, United Kingdom; UK Vaccine Innovation Pathway, National Institute for Health and Care Research, London, United Kingdom

**Keywords:** cancer vaccines, individualized therapies

## Abstract

Cancer vaccines offer transformative potential in oncology, especially with advances in mRNA technologies. To accelerate advances in cancer vaccine research and capitalize on its COVID-19 vaccine leadership, the UK Government has launched the UK Vaccine Innovation Pathway (VIP) through a partnership between the National Institute for Health and Care Research (NIHR), NHS England, and pharmaceutical partners. Its innovative approaches to trial delivery have enabled a 500% increase in patient recruitment with 33 trials open or recently closed across 85 sites and 11 cancer types. Working alongside the Cancer Vaccine Launchpad (CVLP), which pre-screens patients across 55 NHS sites, VIP offers streamlined trial set-up, decentralized delivery, and equity-focused design. The UK’s approach aligns scientific innovation with rapid clinical deployment, creating a scalable, patient-centred model for delivering individualized therapies. This article outlines the strategic framework, outcomes, and lessons from the VIP and CVLP to inform future national strategies in cancer vaccine deployment.

Providing effective treatments for cancer remains a huge unmet need in the world. Traditional therapeutic strategies, such as surgery, chemotherapy, radiotherapy, molecularly targeted agents and immunotherapy, have achieved great advances. However, improvements in care with these treatments are now incremental rather than paradigm changing so new approaches are sought.

The term ‘cancer vaccines’ encompasses a range of agents, which act to up-regulate the patient’s own immune system to recognize and remove cancer cells. Cancer vaccines have been used effectively in the true prophylactic sense to inoculate against cancer-causing viruses such as human papillomavirus (HPV) [1]. Cancer vaccines are also used therapeutically, with adjuvant or palliative intent, as either personalized bespoke cancer vaccines or to target common tumour antigens that are likely to be present in a particular tumour type (‘off the shelf’ vaccines). Whilst cancer vaccines can be made from different backbones, such as using bacterial vectors or dendritic cells, it is currently those similar to COVID-19 vaccine mRNA technology, which are of greatest interest to patients and clinical teams.

Cancer vaccination as a therapeutic strategy is not a new concept. The cell-based cancer vaccine sipuleucel-T (Provenge) was approved for use in the USA [2]. However, sipuleucel-T was not approved by the UK National Institute for Clinical Excellence (NICE) as it was not cost-effective, and so has not become a standard of care. Vaccines using mRNA technology are now being assessed for multiple cancer indications including melanoma, colorectal cancer, and pancreatic cancer [3–5].

Launched in February 2023, the Vaccine Innovation Pathway (VIP) was established to provide a national, cross-sector infrastructure to support the rapid set-up and delivery of vaccine trials in oncology and infectious disease. Designated a Clinical Trial Delivery Accelerator by the UK Government in November 2023, the VIP is embedded within the broader UK clinical research vision to make the country one of the best places in the world to conduct research.

The VIP operates through five integrated pillars (Fig. 1): access to national clinical leadership, optimized research infrastructure, streamlined trial set-up, innovative trial delivery models, and patient centric and inclusive approaches. This structured approach ensures that cancer vaccine trials benefit from a high-functioning ecosystem where sponsors, investigators, and health systems are aligned around speed, quality, and equity.

**Figure 1.**
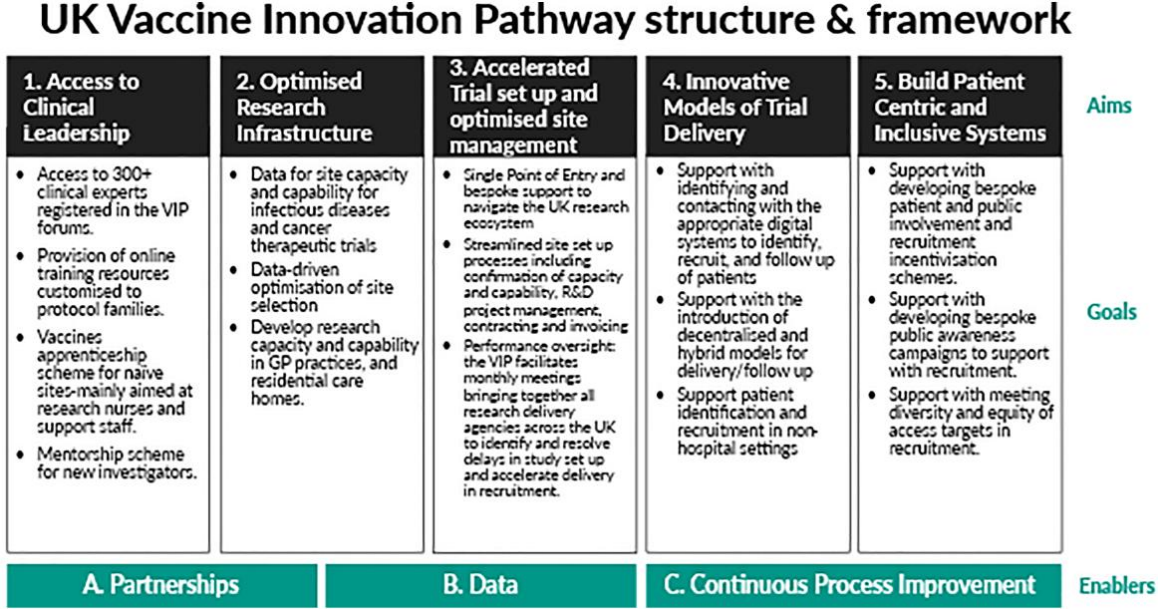
UK vaccine innovation pathway.

## Access to clinical leadership

One of the VIP’s early innovations was the formation of the UK Cancer Vaccine Research Forum. This multi-disciplinary platform brings together clinicians, researchers, delivery teams, and patients to co-design solutions to trial delivery challenges. It offers sponsors rapid feedback on trial design, aligns protocols with UK standard of care, and promotes transparency across the pipeline. The forum has also become a hub for education, with tailored resources developed for patients and healthcare professionals to explain the science and logistics of cancer vaccines.

## Optimized research infrastructure

A core function of the VIP is to ensure that UK sites are ‘vaccine ready’—able to deliver complex cancer vaccine trials efficiently and safely. This involves careful site selection, ensuring that the right investigators, facilities, and support structures are in place. Cancer vaccines, particularly those based on mRNA, are classified as advanced therapy investigational medicinal products (ATIMPs), and their delivery requires specialist pharmacy and regulatory capabilities.

The VIP has conducted extensive mapping of site capabilities across the NHS, including assessments of apheresis availability (critical for early phase immune response monitoring) and pharmacy capacity for mRNA handling. To close gaps in infrastructure, the VIP partnered with Moderna to establish the Vaccine Innovation Fund, which offers targeted funding to support workforce training, equipment upgrades, and regulatory innovation. These investments have enabled more sites to participate in cancer vaccine research, expanding geographic reach and patient access.

## Accelerated clinical trial set-up and optimized site management

The VIP also addresses one of the major historical barriers to commercial trial delivery in the UK: complex and inconsistent trial set-up processes. By acting as a single point of entry for sponsors, the VIP reduces duplication, accelerates key decisions, and ensures that trials move from feasibility to activation without unnecessary delay.

A critical enabler of this has been the National Contract Value Review, led by NHS England, which standardizes costing and contracting across NHS sites. Whilst this has been applied across commercial trials across England, this has been particularly successful for cancer vaccine trials when combined with regular joint meetings between sponsors, investigators, and the VIP team. This coordinated approach has dramatically reduced trial set-up times, helping the UK outperform its benchmarks for cancer vaccine delivery.

## Innovative models of trial delivery

Beyond operational efficiency, the VIP champions new approaches to how trials are delivered. This includes support for remote consent, virtual monitoring, and decentralized delivery models such as mobile research units and hub-and-spoke networks. These innovations reduce participant burden, enable faster recruitment, and make trials more accessible to people who may not live near major cancer centres. In order for such approaches to work, cancer vaccine research must be viewed favourably by both patients and clinical teams, which is largely the case in the UK.

Patients can now explore nearby research opportunities through the National Institute for Health and Care Research (NIHR) ‘Be Part of Research’ online platform, which has become a trusted entry point for the public. Moreover, adaptive and platform trial designs are being encouraged, enabling trials to evolve in response to emerging data and reducing the number of patients needed to achieve meaningful outcomes.

## Build patient centric and inclusive systems

During the pandemic, COVID-19 vaccination rates and research engagement were high across the UK and, as a result, vaccine hesitancy is less pronounced than in some other countries. In the UK, there have been numerous positive media articles on patients receiving cancer vaccines, which have led to interest from the public. Patient involvement in trial design and delivery is vital. The VIP monitors where and how sites are recruiting and works with the public, companies and investigators to improve diversity and equity of access to trials.

**Figure 2.**
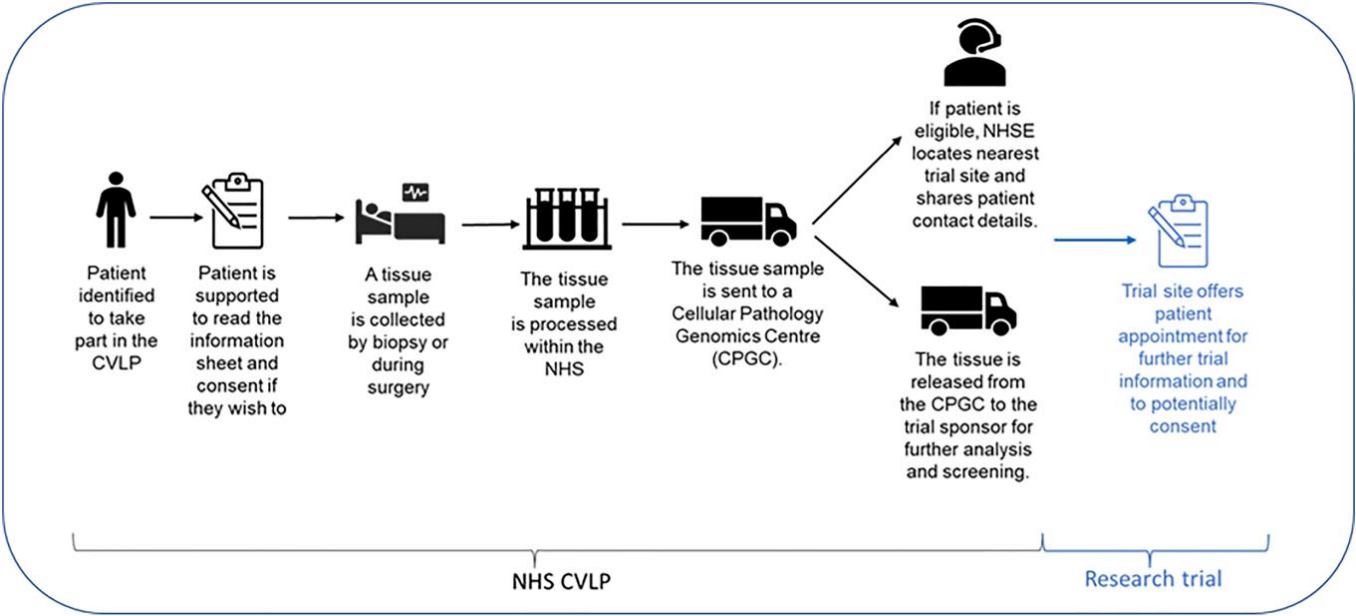
Pathway for CVLP.

Another key component for UK cancer vaccine research is pre-screening patients using the Cancer Vaccine Launchpad (CVLP; Fig. 2). The CVLP identifies suitable patients early in their cancer journey across a wide range of sites, ensuring timely access to investigational vaccines. It was set-up by NHS England and works in parallel with the existing NHS Genomic Medicine Service. The first clinical trial supported by the CVLP was a colorectal cancer vaccine trial sponsored by BioNTech [4]. Between September 2023 and February 2025, 55 CVLP sites screened 3125 patients resulting in 432 eligible patients being referred to the clinical trial. To date, 95% of tissue samples were prepared in the required time frame, with the average time for preparation being 2.5 days. Around 60% of patients in England undergoing colorectal surgery now have access to the trial via the CVLP, resulting in the UK screening over four times the global average for the trial. The CVLP is now pre-screening patients for both a head and neck cancer and a melanoma trial with plans for more.

In partnership with the NIHR, NHS England, and pharmaceutical partners, the above measures have resulted in increases in both the number of cancer vaccine trials and the number of patients recruited to them. As of June 2025, 1937 patients had been recruited to 33 NIHR-portfolio trials running in 85 sites. Recruitment to cancer vaccine trials has increased by 500% in 1 year. There are open studies in 11 cancer indications.

The next phase of delivery will focus on increasing the number of vaccine-ready sites and formalizing referral pathways between research-supporting and research-delivering centres. It will also support the first UK trials focused on cancer prevention in high-risk populations (e.g. individuals with the hereditary cancer syndrome and Lynch Syndrome). The VIP will broaden its remit to include non-mRNA vaccine technologies and explore applications beyond oncology, including neurological and infectious diseases.

In conclusion, the UK has established a world-leading model for the delivery of cancer vaccine trials at scale. Through the coordinated efforts of the VIP and the CVLP, the nation has translated the momentum of COVID-19 vaccine success into a sustainable, scalable infrastructure for individualized cancer therapies. This achievement is not simply about faster trial set-up or higher recruitment—it reflects a deeper shift towards a system where innovation, equity, and patient need are central to research design and delivery. As cancer vaccines move closer to regulatory approval and routine use, the UK’s readiness will mean that UK patients can be amongst the first to benefit.

## Data Availability

The data underlying this article are available in the article.
